# Functional Outcomes of Bicolumnar Plating in Schatzker Type V and VI Proximal Tibial Fractures: A Prospective Study

**DOI:** 10.7759/cureus.86399

**Published:** 2025-06-19

**Authors:** Ajayguru Senthurvelan, Ashok Nayak, Ravikumar Biradar, Anil Bulagond, Prashant Kenganal, Srikant Kulkarni, Sahebagouda Patil

**Affiliations:** 1 Department of Orthopedics, Shri B. M. Patil Medical College, Hospital and Research Centre, Bharatiya Lingayat Development Educational Association (Deemed to be University), Vijayapura, IND

**Keywords:** bicolumnar plating for tibia, bicondylar tibial plateau fractures, dual locking plates, functional outcome of tibial plateau fractures, modified rasmussen score, proximal tibia fracture orif with plating, schatzker type v and vi

## Abstract

Background

Managing bicondylar intra-articular tibial plateau fractures resulting from high-energy trauma remains challenging, primarily due to complex fracture morphology and significant associated soft tissue injury, which contribute to a high risk of complications. Despite advancements in management, outcomes for unstable bicondylar fractures remain suboptimal. This study aimed to evaluate the functional outcomes and complications associated with internal fixation of bicondylar tibial plateau fractures using dual plating through two incisions.

Objectives

To assess the functional outcomes of surgical treatment for proximal tibial fractures (Schatzker types V and VI) managed with bicolumnar plating.

Materials and methods

A prospective clinical study was conducted from April 2023 to December 2024 at Shri B.M. Patil Medical College, Hospital and Research Centre, Vijayapura. Thirty-one patients with Schatzker type V and VI proximal tibia fractures treated with bicolumnar plates were included. Functional outcomes were evaluated using the Modified Rasmussen Score at two weeks, six weeks, three months, and six months postoperatively.

Results

Significant improvement in Modified Rasmussen Scores was observed at all postoperative time points. All fractures achieved union with a mean time to union of 13.75 weeks. The average knee range of motion (ROM) ranged from 1.5° extension lag to 130° flexion (range: 0°-10° extension lag; 100°-135° flexion). The mean final Rasmussen functional score was 25.48. Postoperative radiographs showed a mean medial proximal tibial angle (MPTA) of 87.51° (range 82°-92°) and a mean posterior proximal tibial angle (PPTA) of 8.13° (range 4°-14°). Complications were noted in nineteen patients, but there were no cases of secondary loss of reduction, implant failure, malunion, or nonunion.

Conclusion

Dual locking plate fixation for bicondylar tibial plateau fractures offers a reliable and effective treatment strategy, ensuring rigid stabilization and facilitating favorable functional and radiological outcomes. Bicolumnar plating has proven to be particularly effective in the management of Schatzker type V and VI fractures, yielding excellent results when performed by skilled surgeons. Early rehabilitation, including the use of continuous passive motion (CPM), plays a crucial role in minimizing postoperative complications such as knee stiffness and promoting optimal joint mobility.

## Introduction

Tibial plateau fractures encompass a broad spectrum of injuries, from simple, low-energy patterns to complex, high-energy fractures. Schatzker types V and VI are classified as unstable bicondylar injuries and are typically associated with significant articular depression, comminuted condylar fragments, metaphyseal-diaphyseal extension, and marked soft tissue compromise [1,2]. The primary goals of treatment include the preservation of soft tissue integrity, restoration of joint congruity, and maintenance of anatomical alignment to optimize functional recovery [3].

Conservative management methods, such as traction or casting, were historically common but often led to complications like joint stiffness and malunion [4]. The advent of external fixation, including hybrid systems, provided an option for early stabilization with limited soft tissue damage. However, these techniques were often complicated by pin tract infections, septic arthritis, and patient non-compliance [5,6].

Contemporary fixation strategies employ locking plate systems and minimally invasive plate osteosynthesis (MIPO), improving both biomechanical stability and soft tissue preservation. Although single lateral locking plates can provide sufficient fixation in selected cases, they may not adequately address medial instability, particularly in the presence of coronal fractures [7,8].

Dual plating through a two-incision technique allows for rigid fixation of both columns, reducing the risk of varus collapse and improving fracture visualization. Despite its advantages, this approach involves extensive soft tissue dissection and carries a higher risk of wound-related complications [9-11].

This study aims to evaluate the clinical and radiological outcomes of Schatzker type V and VI tibial plateau fractures treated with dual plating via a two-incision approach and to assess the complication rates associated with this technique.

## Materials and methods

This prospective clinical study was conducted in the Department of Orthopedics at Shri B.M. Patil Medical College, Hospital and Research Centre, BLDE (Deemed to be University), Vijayapura, from April 1, 2023, to December 1, 2024. Patients aged 18 years and older presenting with Schatzker type V and VI proximal tibial fractures were evaluated clinically and radiologically for inclusion. Only closed fractures and Gustilo-Anderson type I open fractures sustained within three weeks of injury were considered eligible. Patients with pathological fractures, compound fractures classified as Gustilo-Anderson type II or III, neurovascular compromise, or ipsilateral lower limb fractures were excluded. Informed written consent was obtained from all participants prior to inclusion.

Preoperative management 

**Figure 1:**
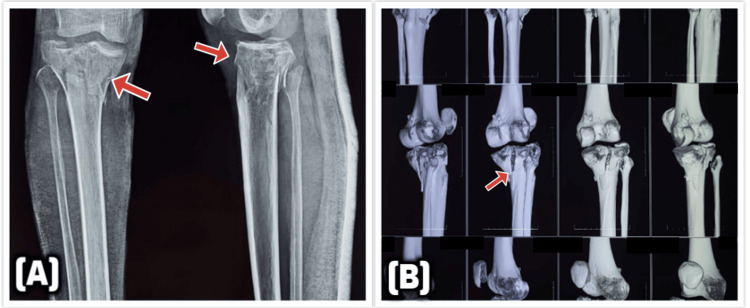
Preoperative radiographic and CT evaluation of a bicondylar tibial plateau fracture (A) AP and lateral radiographs showing a bicondylar tibial plateau fracture with depression and displacement of both medial and lateral condyles (red arrows). (B) 3D-CT reconstructions further delineate the fracture morphology, comminution, and joint surface involvement, aiding in surgical planning (red arrow indicates fracture line). CT: computed tomography; AP: anteroposterior

All patients underwent standard preoperative imaging, including anteroposterior (AP) and lateral radiographs of the affected knee, as well as computed tomography (CT) scans with three-dimensional reconstruction to assist with surgical planning, as depicted in Figure 1.

Upon admission, the injured limb was reduced with gentle traction, immobilized using skeletal traction, and placed on a Bohler-Braun splint in an elevated position. In patients presenting with tense knee joint effusion, aspiration was performed under aseptic conditions. Patients were monitored regularly for any signs of compromised peripheral circulation or neurological deficits. Prophylactic intravenous antibiotics, ceftriaxone 1 g and amikacin 500 mg, were administered upon admission and repeated preoperatively. Surgical fixation was undertaken only after careful evaluation of the skin and soft tissue condition. The presence of contusions or fracture blisters was considered indicative of severe soft tissue compromise, in which case surgery was deferred until the resolution of swelling and the reappearance of skin wrinkling. In instances where soft tissue recovery was inadequate despite observation, patients were managed with a ring fixator and excluded from the study.

All procedures were performed by the same senior surgeon under general or spinal anesthesia. While the Schatzker classification system was used to categorize the fractures, the Three-Column classification was additionally utilized for fractures with posterior column involvement or complex patterns not adequately described by Schatzker alone [12]. Traction radiographs or CT scans were employed preoperatively to better delineate the fracture morphology, which was often simplified by ligamentotaxis.

Surgical procedure

**Figure 2:**
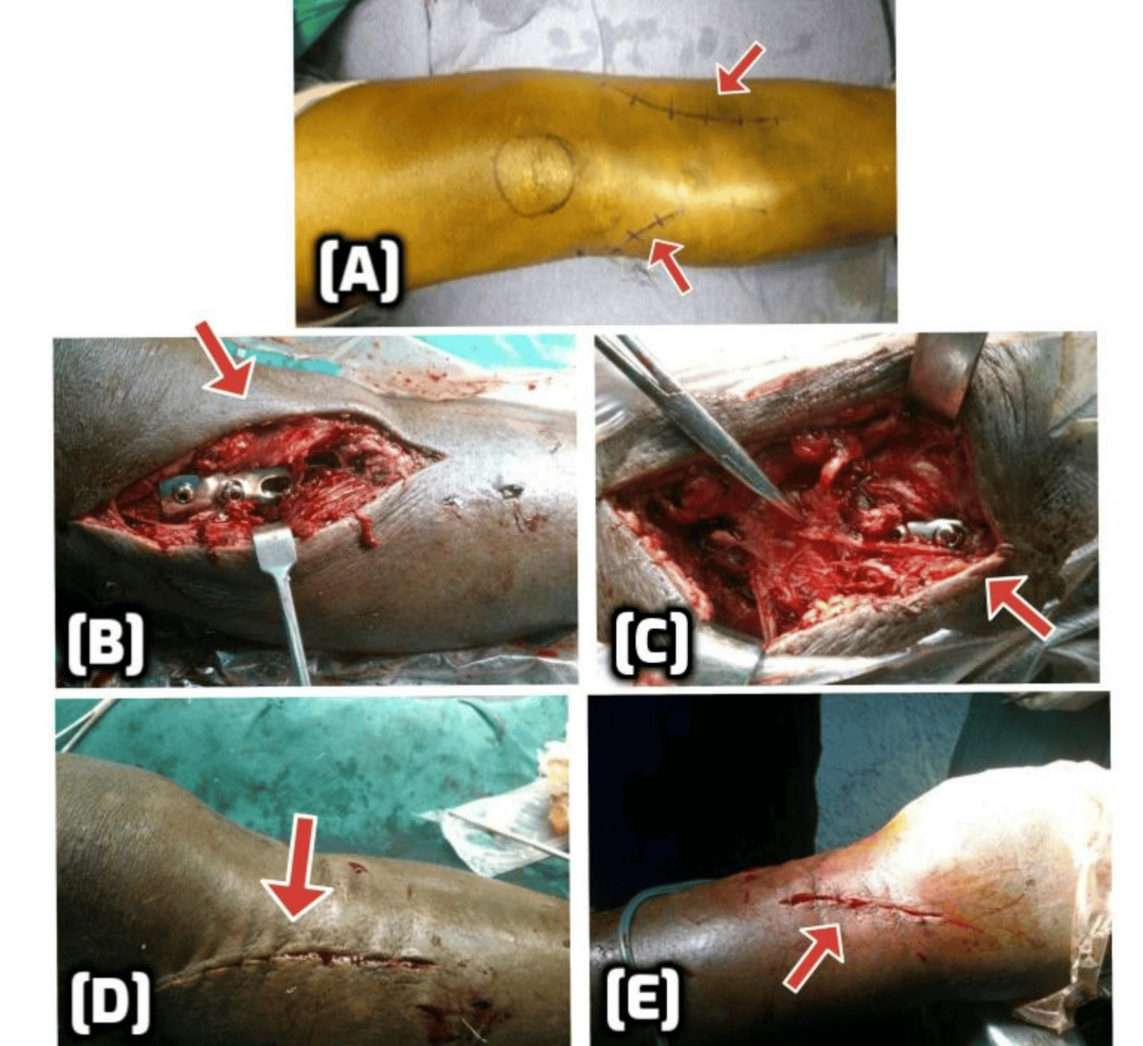
Intraoperative steps of dual incision and bicolumnar fixation for Schatzker type VI tibial plateau fracture (A) Preoperative markings for the anterolateral and posteromedial approaches (red arrows). (B) Medial column fixation through the posteromedial approach with a medial locking plate. (C) Intraoperative image showing fixation of the lateral condyle using a lateral locking plate via the anterolateral approach. (D) Closure of the medial wound. (E) Closure of the lateral wound with a suction drain in situ.

All patients were positioned supine on a radiolucent operating table under spinal or epidural anesthesia. A folded pillow was placed beneath the knee to facilitate slight flexion, and a pneumatic tourniquet was applied. The procedure was performed under fluoroscopic guidance using a C-arm image intensifier to confirm joint congruity and fracture reduction intraoperatively. The surgical approach was staged, beginning with the posteromedial (medial) column, which was typically addressed first, as depicted in Figure 2. A longitudinal incision approximately 2 cm posterior to the posteromedial border of the tibial shaft was made. After blunt dissection, the pes anserinus was retracted anteriorly, and the fascia overlying the gastrocnemius was incised. In cases with intra-articular involvement, a sub-meniscal arthrotomy was performed to visualize the fracture line. Articular fragments were mobilized in knee flexion and external rotation, and any depressed segments were elevated using a bone punch. If required, autologous cancellous bone grafts were placed in the metaphyseal voids. The posteromedial fragment was reduced and stabilized using a contoured 3.5 mm or 4.5 mm proximal tibial T- or L-buttress plate, along with compression screws oriented in a posteroanterior direction.

The lateral column was approached through an anterolateral or "lazy-S" incision centered over Gerdy’s tubercle, extending proximally and distally as needed. After sub-meniscal arthrotomy, lateral articular fragments were elevated using a chisel or bone punch to correct depression. The lateral plateau was then anatomically reduced and temporarily stabilized using percutaneous K-wires. A pre-contoured proximal tibial lateral locking compression plate (LCP) was used to fix the lateral condyle, providing a buttress to prevent lateral collapse. A large compression clamp was employed in some cases to maintain reduction during definitive fixation.

**Figure 3:**
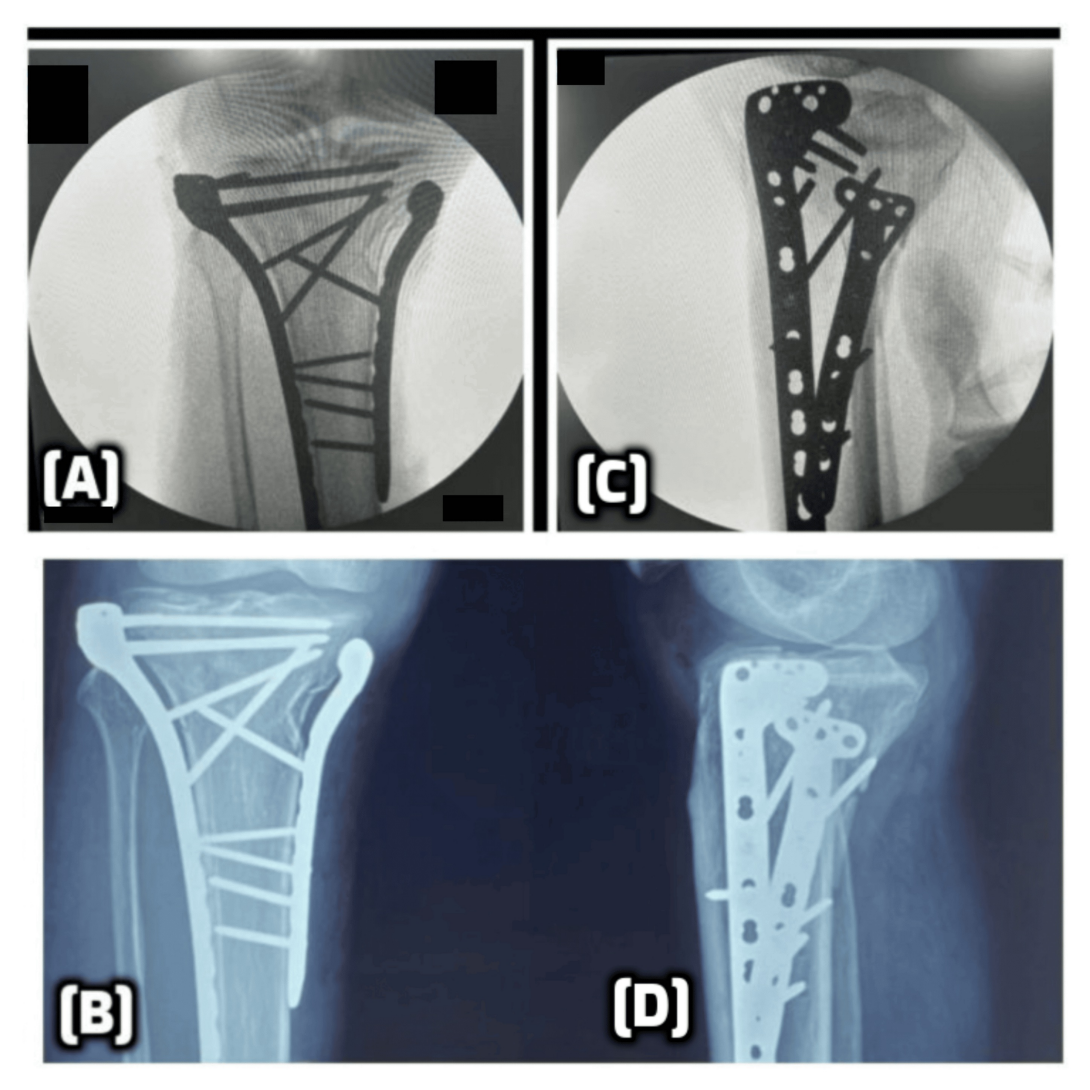
Intraoperative and postoperative radiographic views following dual plating of a bicondylar tibial plateau fracture (A, C) Intraoperative fluoroscopic images showing fixation of a bicondylar tibial plateau fracture using a dual plating technique via a two-incision approach. (B, D) Immediate postoperative radiographs in AP and lateral views demonstrate anatomical reduction and stable fixation with lateral and medial LCP. LCP: locking compression plates; AP: anteroposterior

Once both columns were fixed, the fracture reduction and hardware positioning were confirmed under fluoroscopy, as depicted in Figure 3. Wounds were irrigated and closed in layers. A posterior above-knee splint or back-slab was applied postoperatively to protect the repair during the initial recovery phase.

Postoperative management 

**Figure 4:**
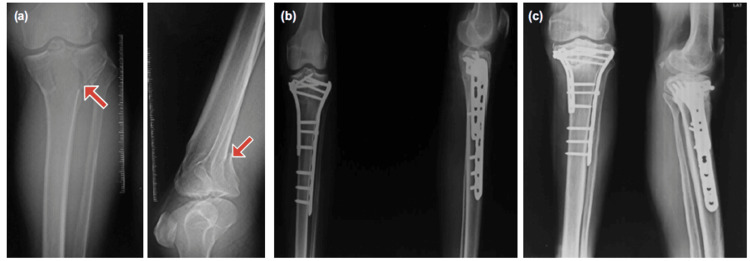
Radiographic evaluation of Schatzker type V tibial plateau fracture treated with dual plating (A) Preoperative AP and lateral radiographs showing a bicondylar tibial plateau fracture (Schatzker type V) involving both medial and lateral condyles (red arrows). (B) Immediate postoperative radiographs demonstrating anatomical reduction and internal fixation using dual locking plates via a two-incision approach. (C) Six-month follow-up radiographs revealed maintained reduction, implant position, and progressive fracture healing. AP: anteroposterior

**Figure 5:**
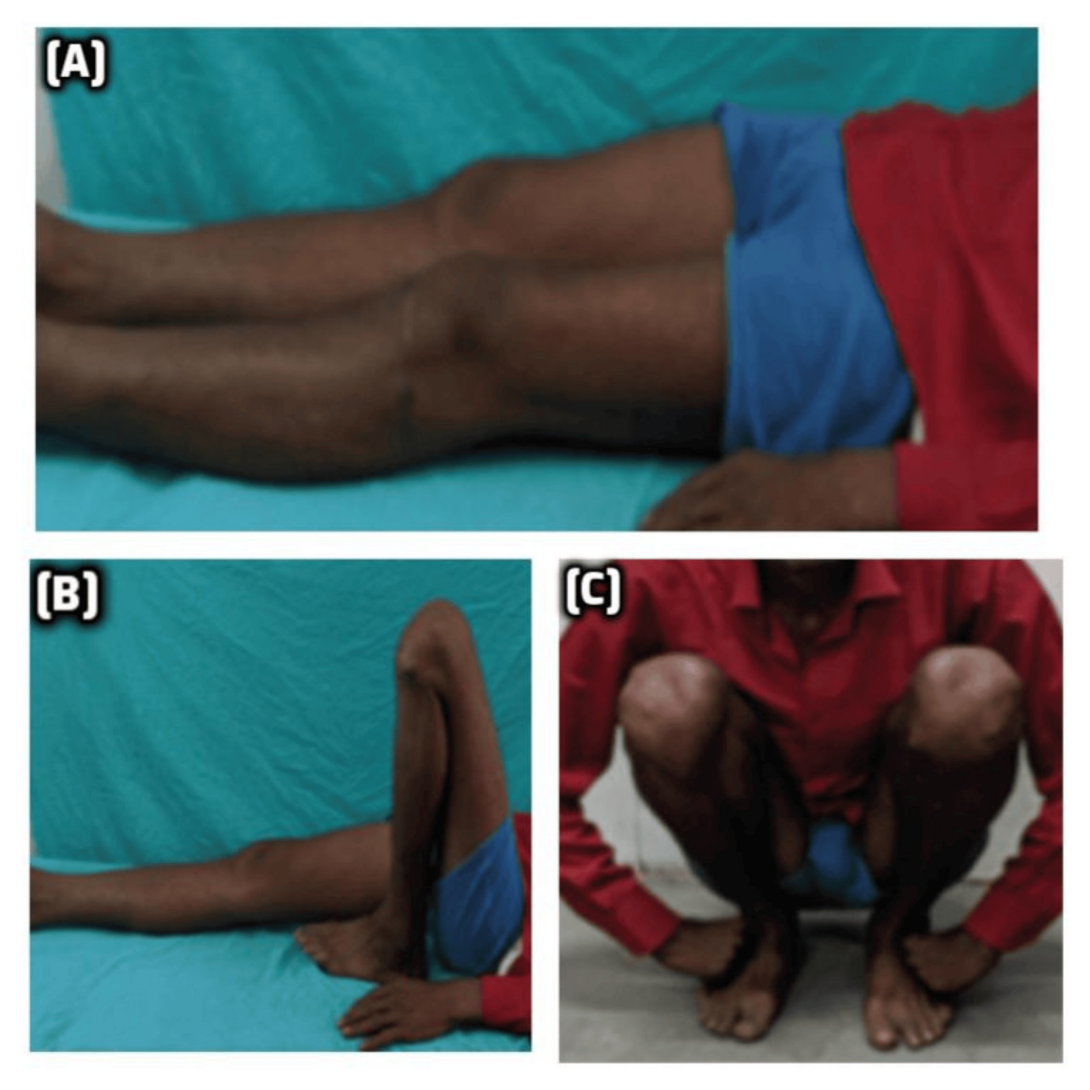
Postoperative functional recovery following dual plating for Schatzker type V tibial plateau fracture (A) Clinical photograph showing full extension of the knee in the prone position. (B) Near-complete flexion of the knee demonstrates good ROM. (C) The patient was able to perform a full squat, indicating an excellent functional outcome. ROM: range of motion

All patients followed a standardized postoperative rehabilitation protocol. Isometric quadriceps and hamstring exercises, along with passive knee mobilization, were initiated on the second postoperative day. Partial weight-bearing was permitted between the sixth and eighth week, with progression to full weight-bearing between the 12^th^ and 14^th^ week, contingent upon radiological evidence of fracture union. Follow-up evaluations were conducted at the second weeks, sixth weeks, third months, and sixth months postoperatively, as depicted in Figures 4, 5. Functional outcomes were assessed using the Modified Rasmussen’s Scoring System, originally described by Rasmussen [13]. The scoring system evaluates pain, walking capacity, extension, range of motion (ROM), and stability, with a maximum score of 30. The results were categorized as Excellent (27-30), Good (20-26), Fair (10-19), and Poor (<10).

Radiological Assessment

**Figure 6:**
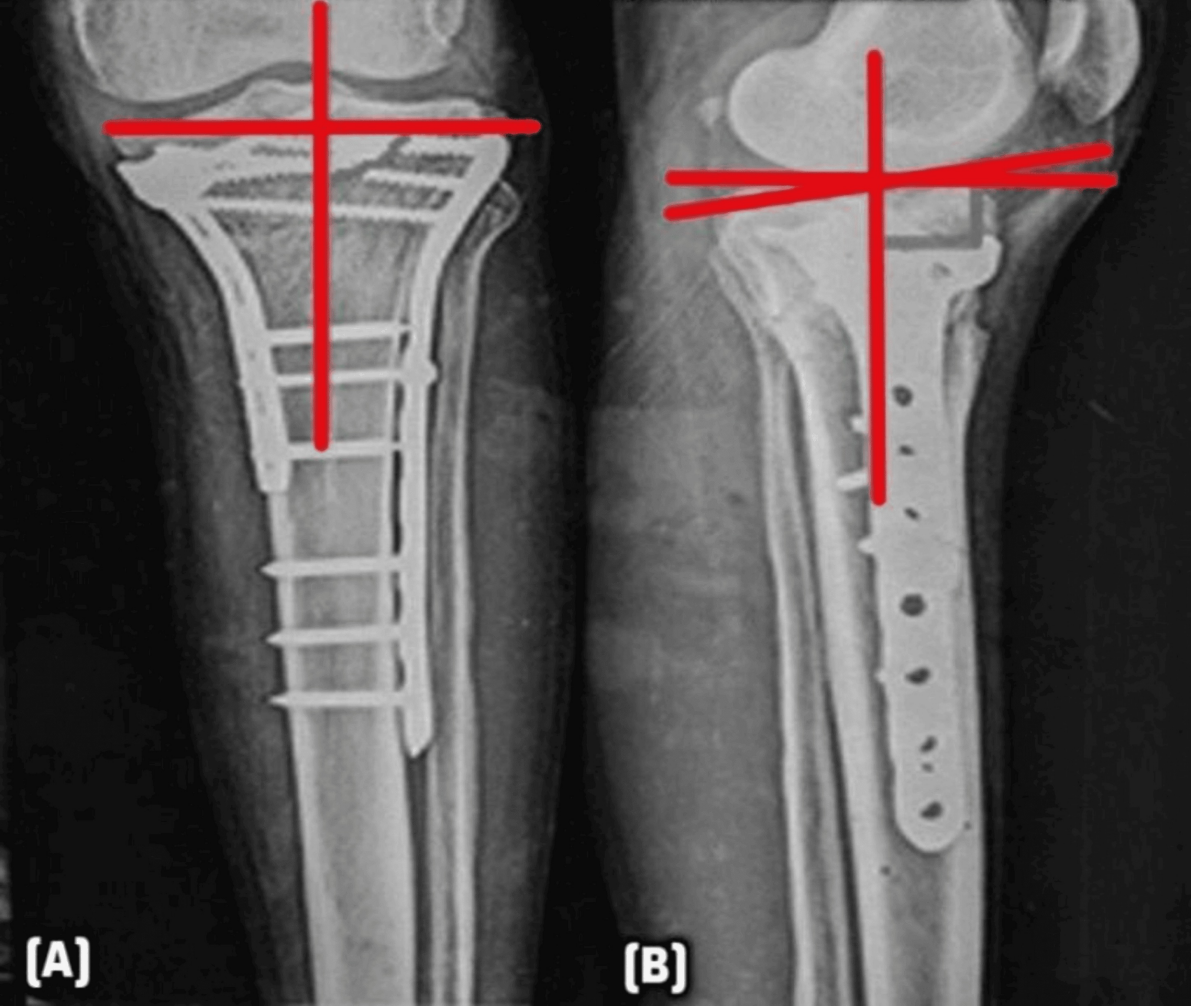
Radiographic assessment of postoperative alignment using MPTA and PPTA (A) Postoperative AP radiograph showing the MPTA measured between the tibial mechanical axis and the articular surface of the proximal tibia. The observed angle is within the normal range of 82°-92°. (B) Lateral radiograph showing the PPTA, formed between the tibial shaft and the tibial plateau, with a normal reference range of 4°-14°. These angles help assess coronal and sagittal alignment after surgical fixation of proximal tibial fractures. MPTA: medial proximal tibial angle; PPTA: posterior proximal tibial angle; AP: anteroposterior

Postoperative radiographic evaluation was conducted to assess both joint congruity and limb alignment using standardized AP and lateral radiographs. Joint congruity was analyzed by measuring the intra-articular step-off (depression) on AP views, with a step-off greater than 2 mm considered indicative of mal-reduction. All measurements were taken on calibrated radiographs to ensure accuracy. Limb alignment was evaluated in both the coronal and sagittal planes using the medial proximal tibial angle (MPTA) and the posterior proximal tibial angle (PPTA), respectively, as illustrated in Figure 6.

Additional radiographic parameters recorded included restoration and maintenance of the joint line, evidence of infection, delayed or non-union, and signs of fracture collapse. Fracture union was confirmed by the presence of bridging callus across at least three cortices on AP and lateral radiographs, in conjunction with pain-free full weight-bearing.

Statistical analysis

Data were entered in Microsoft Excel (Microsoft Corporation, Redmond, USA) and analyzed using IBM SPSS Statistics for Windows, Version 20.0 (IBM Corp, Armonk, USA ). Descriptive statistics were calculated for all variables. Continuous variables were expressed as mean ± standard deviation and categorical variables as frequencies and percentages. For variables with both preoperative and postoperative values within the same group, comparisons were made using paired t-tests. Chi-square tests were used for categorical variables where applicable. A p-value of <0.05 was considered statistically significant.

## Results

**Figure 7:**
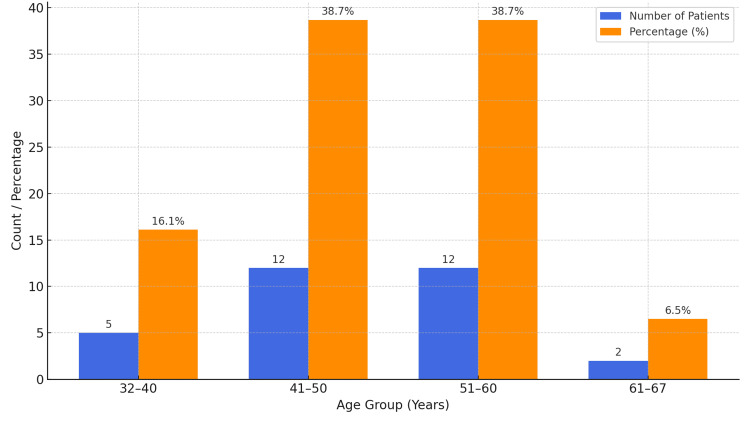
Age distribution

**Figure 8:**
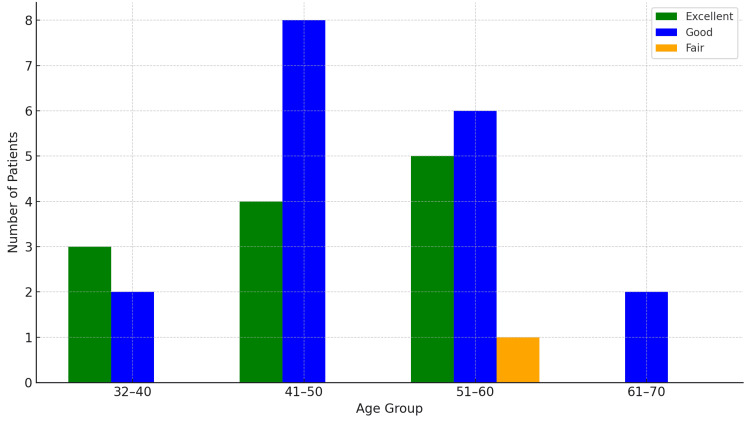
Bar graph showing the distribution of Modified Rasmussen Score gradings (Excellent, Good, and Fair) across different age groups

A total of 31 patients with Schatzker type V and VI proximal tibial fractures were included in the study, with a mean age of 49.6 years (range: 32-67 years), as depicted in Figure 7. The distribution of functional outcomes based on age group is presented in Figure 8 and Table 1.

**Table 1:** Results based on age distribution

SI. no.	Age group	Number of patients	Average Modified Rasmussen Score		Grading		
				Excellent	Good	Fair	Poor
1	32-40	5	26.60	3	2	0	-
2	41-50	12	25.58	4	8	0	-
3	51-60	12	25.25	5	6	1	-
4	61-70	2	23.50	0	2	0	-

**Figure 9:**
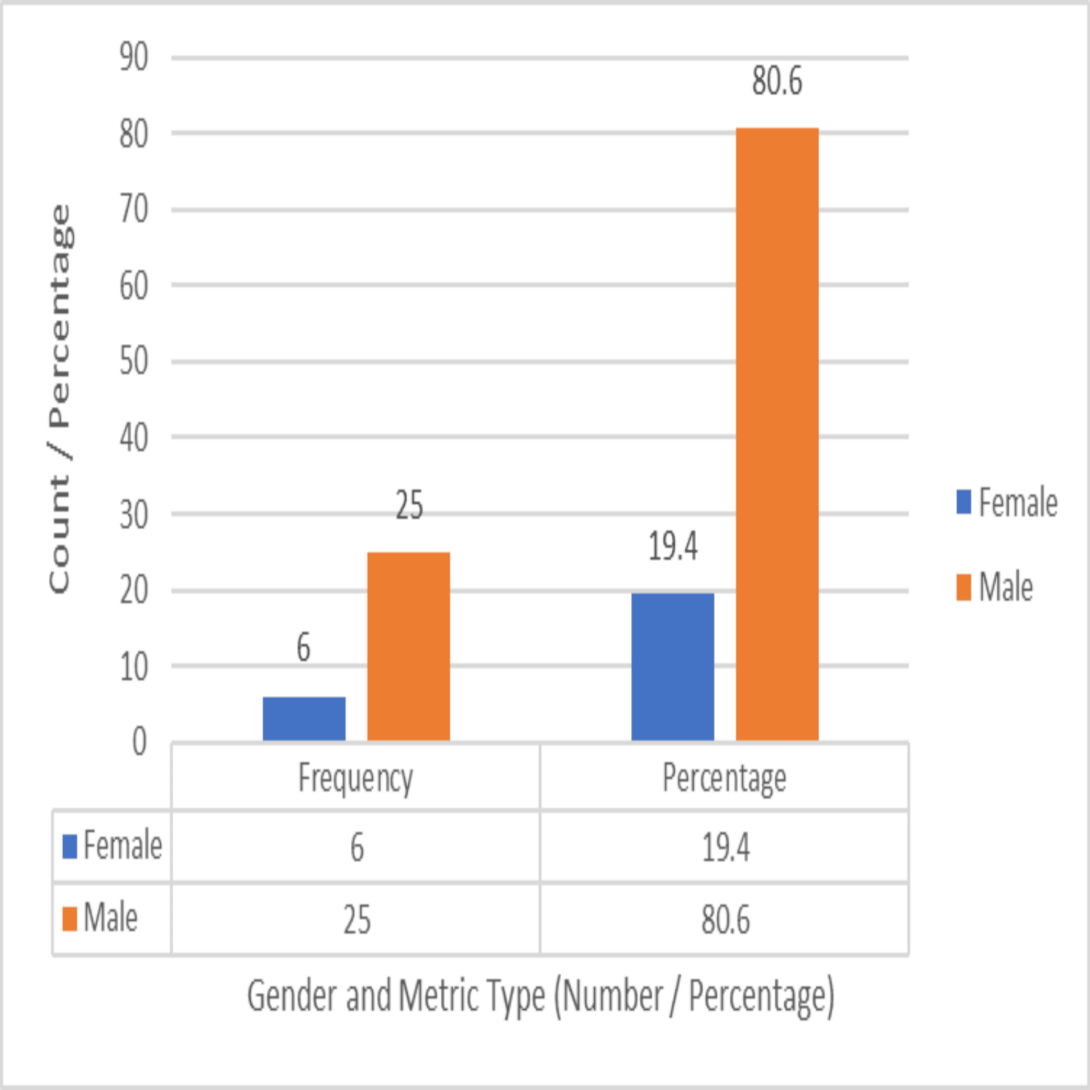
Distribution of sex

The majority were males (n=25, 80.6%), and six were females (19.4%). Male-to-female ratio = 4.2:1, as depicted in Figure 9.

**Figure 10:**
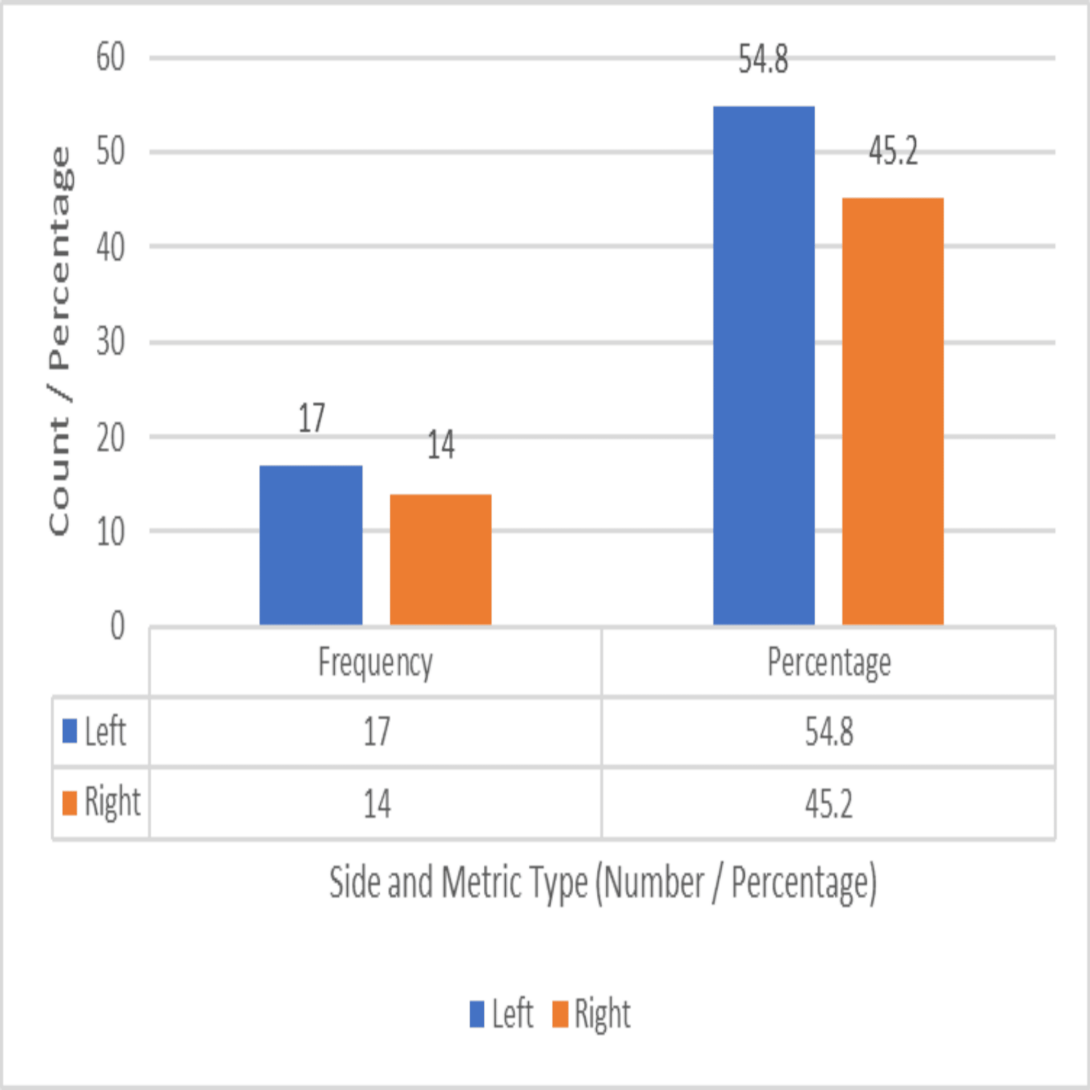
Distribution of side involvement (left vs. right)

The left side was affected in 17 patients (54.8%) and the right side in 14 patients (45.2%), as depicted in Figure 10.

**Figure 11:**
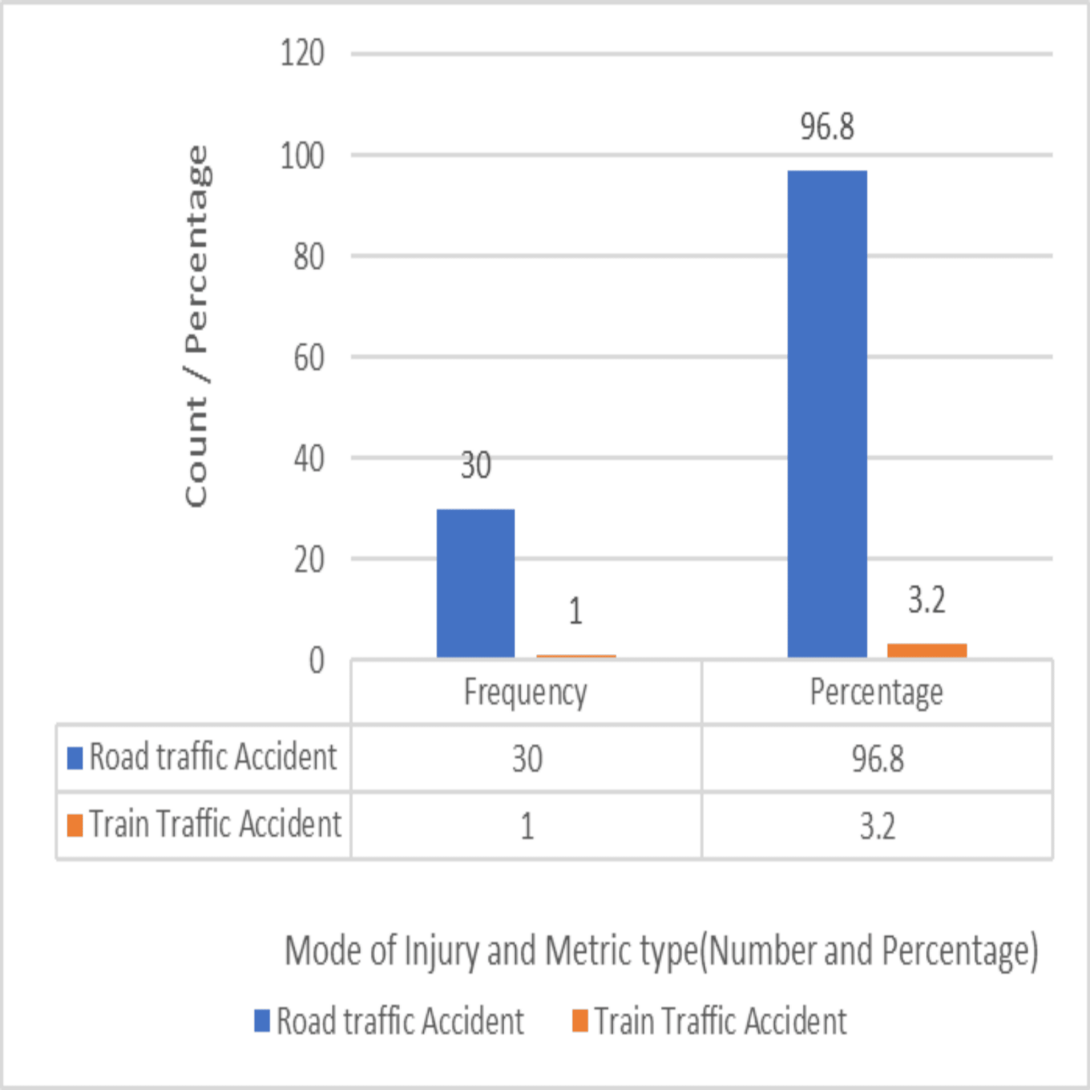
Mode of injury

Road traffic accidents were the predominant mode of injury (n=30, 96.8%), with one case resulting from a train traffic accident (3.2%), which explains the high-energy nature of the injury, as depicted in Figure 11. Every patient who participated in our study experienced significant energy injuries from automobile accidents. None had suffered minor trauma-related injuries. Low-energy injuries are likely to cause complicated tibial plateau fractures in elderly people with osteoporotic bones. But in our series, we have not encountered any such cases.

**Figure 12:**
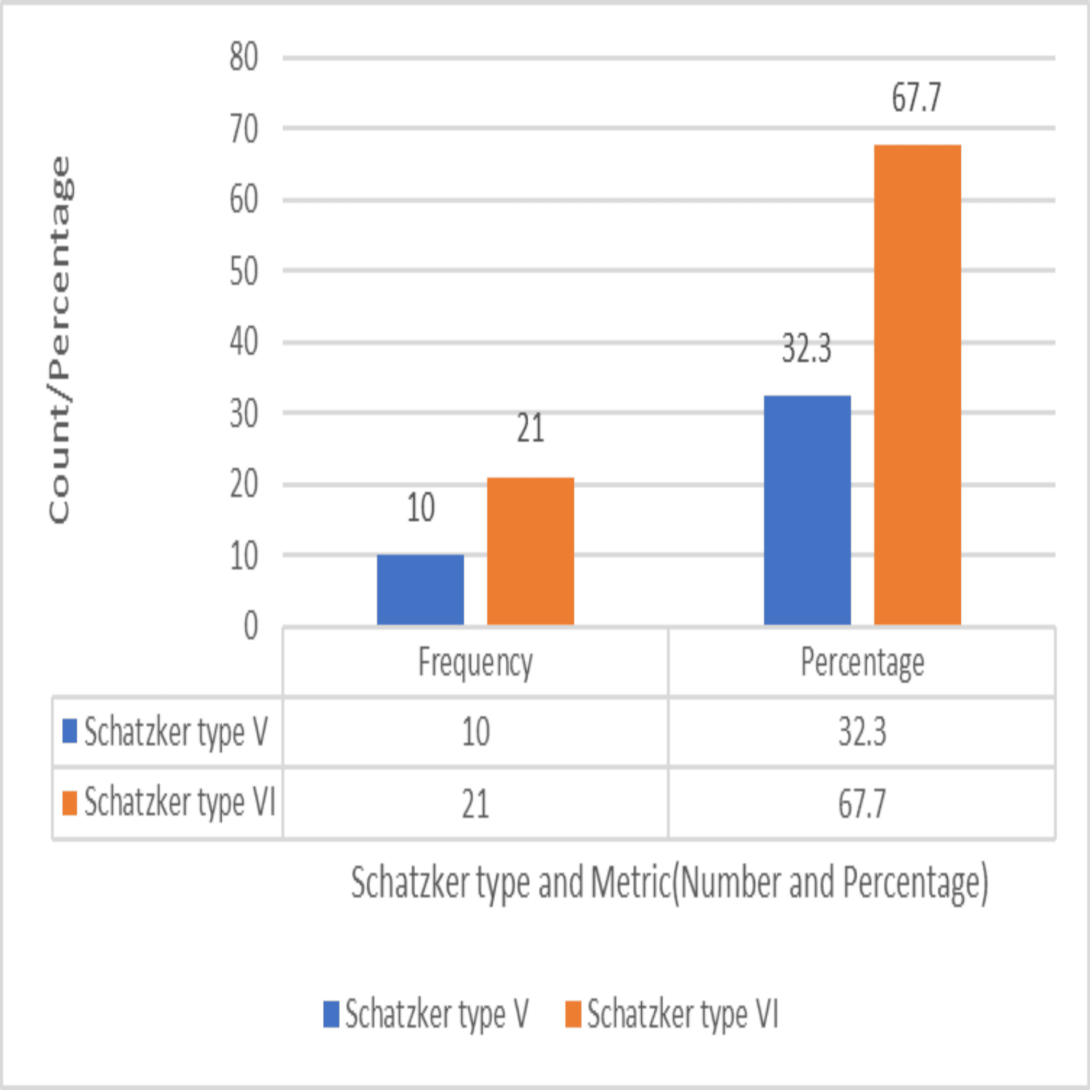
Distribution based on Schatzker type

About 24 patients had solitary tibial plateau fractures, while seven patients (22.6%) had associated injuries, which included ipsilateral clavicle fractures in three patients, ipsilateral distal radius fractures in two patients, contralateral lateral malleolus fracture in one patient, and a contralateral both bone leg fracture with an associated shaft of femur fracture in one patient. The delay before surgery ranged from three to 20 days, with a mean of 11.2 days. Schatzker type V fractures were seen in 10 patients (32.3%) and type VI in 21 patients (67.7%), as depicted in Figure 12.

**Figure 13:**
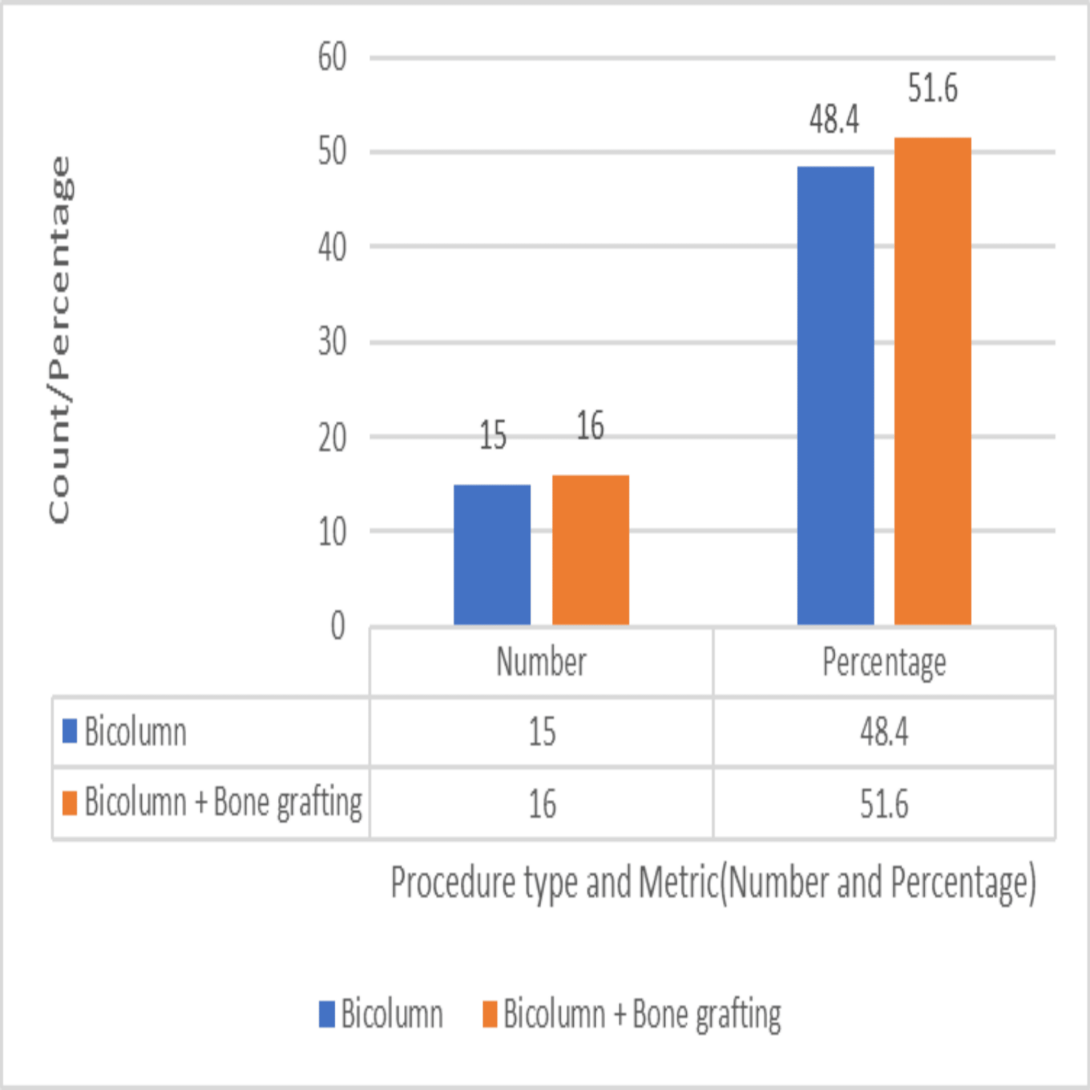
Distribution of patients treated with bicolumnar plating alone versus those who additionally required bone grafting

All patients received routine surgery, which included anterolateral and posteromedial incisions. All patients underwent bicolumnar plating, with 15 patients (48.4%) treated using dual plates alone and 16 patients (51.6%) requiring additional autologous bone grafting, as depicted in Figure 13. The need for bone grafting likely reflects the extent of metaphyseal comminution or bone voids encountered intraoperatively.

**Figure 14:**
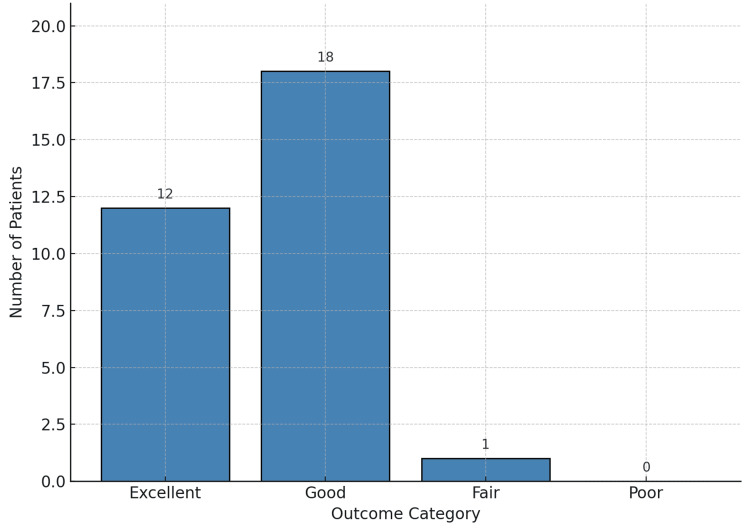
Distribution of patients according to the Modified Rasmussen’s functional outcome scores

For lateral column fixation, 15 patients (48.4%) received LCPs and 16 patients (51.6%) were treated with buttress plates. Notably, there was no significant difference in functional outcomes between these two implant groups. For medial column fixation, all 31 patients (100%) were treated with buttress plates. The postoperative radiological alignment showed a mean MPTA of 87.51° (range: 85.21°-90.11°) and a mean PPTA of 8.1° (range: 6.66°-10.11°), indicating maintenance of anatomical alignment. Articular step-off was ≤2 mm in 27 patients, while four patients had a step-off of >2 mm (range: 3-5 mm). Of these, three patients had a Modified Rasmussen’s functional score graded as Good, while one patient had a Fair outcome. No patients with Excellent scores demonstrated articular incongruity. The average time to union was 13.9 weeks (range: 11-16 weeks), and the mean follow-up duration was 23.1 weeks (range: 20-26 weeks). Functional assessment using the Modified Rasmussen Score revealed Excellent outcomes (score 27-30) in 12 patients (38.7%), Good outcomes (score 20-26) in 18 patients (58.1%), and Fair outcomes (score 10-19) in one patient (3.2%), with no Poor outcomes, as depicted in Figure 14. The mean Rasmussen score was 25.7±2.5, with 96.8% of patients achieving good to excellent results.

**Figure 15:**
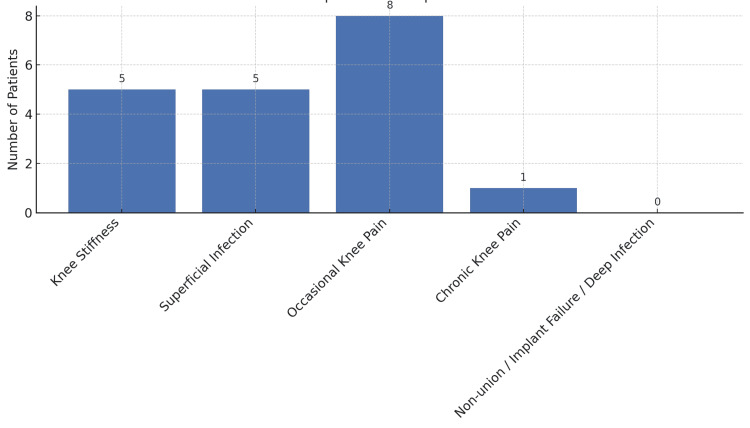
Distribution of postoperative complications among 31 patients who underwent bicolumnar plating for Schatzker type V and VI proximal tibial fractures

Postoperative complications were observed in 19 patients (61.3%), including knee stiffness in 16.1% (5/31) of patients. Among these, two patients (6.5%) had a ROM limited to 10°-80°, two patients (6.5%) had a ROM between 0°-90°, and one patient (3.2%) had a ROM between 10°-90°. Poor compliance with physiotherapy protocols was identified as the primary cause of stiffness. Superficial wound infections were observed in 16.1% (5/31) of patients during the postoperative period. All cases were effectively managed with prompt debridement, regular wound care, and appropriate antibiotic therapy, resulting in satisfactory wound healing and excellent functional recovery. Occasional knee pain was reported by 25.8% (8/31) of patients; however, this did not interfere with daily activities, and was considered a minor complication, all these patients achieved a Good functional outcome based on the Modified Rasmussen Score. Chronic knee pain was noted in one patient (3.2%), who had a Fair functional outcome. No cases of non-union, implant failure, or deep infection were encountered, as depicted in Figure 15. 

Table 2 presents detailed information on 31 patients who underwent bicolumnar plating for Schatzker type V and VI proximal tibial fractures.

**Table 2:** Demographic profile, injury characteristics, surgical details, radiological parameters, and postoperative outcomes in patients treated with bicolumnar plating for Schatzker type V and VI tibial plateau fractures The mechanism of injury was noted as either RTA or TTA. The side injured refers to the laterality of the affected limb. Associated injuries include any additional trauma sustained at the time of the incident. The time delay before surgery indicates the interval, in days, from injury to surgical intervention. Fractures were classified according to the Schatzker system (type V or VI). The surgical procedure performed was either bicolumnar plating alone or with BG, when applicable. The implant used for lateral column fixation was either an LCP or a buttress plate. Postoperative alignment was assessed using the MPTA and the PPTA, both measured in degrees. Functional outcomes were evaluated using the Modified Rasmussen’s Score during postoperative follow-up. Radiographic union was assessed based on the time to union (in weeks), and the duration of clinical follow-up was also recorded in weeks. IP no: inpatient number; F: female; M: male; RTA: road traffic accident; TTA: train traffic accident; LCP: locking compression plate; MPTA: medial proximal tibial angle; PPTA: posterior proximal tibial angle; BG: bone grafting

SI. no.	Name	Age (years)	Sex	IP no.	Mode of injury	Side injured	Associated injuries	Time delay before surgery	Schatzker type	Procedure	Lateral column implant	MPTA	PPTA	Mod. Rasmussen score postoperative	Time to union (weeks)	Follow-up (weeks)	Complications
1	Patient.1	58	M	55390	RTA	Left		3 days	VI	Bicolumn	Buttress plate	86.55	8.88	25	15	20	Knee stiffness
2	Patient.2	57	M	56415	RTA	Right		6 days	VI	Bicolumn	Buttress plate	86.81	7.55	26	12	26	Superficial infection
3	Patient.3	35	M	65815	RTA	Left		7 days	V	Bicolumn	LCP	87.22	7.33	26	14	22	Occasional pain
4	Patient.4	51	F	57392	TTA	Left	Right shaft of femur right and right both bone leg fracture	10 days	V	Bicolumn+BG	LCP	85.21	6.66	19	16	26	Chronic knee pain
5	Patient.5	55	M	9573	RTA	Left	Left clavicle fracture	12 days	VI	Bicolumn+BG	LCP	87.33	8.26	27	13	24	
6	Patient.6	63	M	15100	RTA	Right		20 days	VI	Bicolumn+BG	Buttress plate	88.22	7.99	26	13	21	Occasional pain
7	Patient.7	60	M	27539	RTA	Right		17 days	VI	Bicolumn	Buttress plate	86.66	7.11	22	14	20	Knee stiffness
8	Patient.8	45	M	56943	RTA	Left		12 days	V	Bicolumn	Buttress plate	86.99	7.63	24	16	22	Superficial infection
9	Patient.9	42	M	47812	RTA	Right		10 days	VI	Bicolumn	Buttress plate	89.69	9.11	27	12	24	
10	Patient.10	50	F	50178	RTA	Left	Right distal radius fracture	16 days	V	Bicolumn+BG	LCP	86.67	7.43	25	14	25	Occasional pain
11	Patient.11	56	F	30145	RTA	Right		10 days	VI	Bicolumn	LCP	86.22	9.78	27	13	21	
12	Patient.12	47	M	88396	RTA	Right		5 days	V	Bicolumn+BG	Buttress plate	89.11	9.55	28	12	22	
13	Patient.13	67	F	61853	RTA	Right	Left lateral malleoli fracture	19 days	VI	Bicolumn	LCP	85.71	7.11	21	14	23	Occasional pain
14	Patient.14	46	M	84738	RTA	Right		20 days	V	Bicolumn	LCP	87.77	8.11	27	11	25	
15	Patient.15	40	M	93296	RTA	Left		10 days	VI	Bicolumn+BG	Buttress plate	88.88	8.53	27	13	20	
16	Patient.16	32	M	107958	RTA	Left		8 days	VI	Bicolumn+BG	LCP	90.11	9.99	29	12	21	
17	Patient.17	45	M	107668	RTA	Left		6 days	VI	Bicolumn+BG	LCP	89.99	10.11	29	14	24	
18	Patient.18	55	M	109229	RTA	Right		9 days	VI	Bicolumn+BG	LCP	87.31	8.34	27	14	24	
19	Patient.19	52	M	30554	RTA	Right		11 days	V	Bicolumn+BG	LCP	86.66	7.83	25	16	21	Superficial infection
20	Patient.20	58	M	42890	RTA	Left		9 days	VI	Bicolumn+BG	Buttress plate	89.67	8.22	28	15	20	
21	Patient.21	45	M	67992	RTA	Left		10 days	VI	Bicolumn+BG	Buttress plate	88.37	7.81	26	14	23	Occasional pain
22	Patient.22	59	M	55390	RTA	Left		3 days	VI	Bicolumn	Buttress plate	87.99	7.33	26	15	20	Knee stiffness
23	Patient.23	46	M	56415	RTA	Right		6 days	V	Bicolumn	Buttress plate	86.34	7.21	25	12	26	Superficial infection
24	Patient.24	49	M	65815	RTA	Left		7 days	VI	Bicolumn	LCP	87.32	8.01	26	14	22	Occasional pain
25	Patient.25	50	F	57392	RTA	Left	Left clavicle fracture	10 days	VI	Bicolumn+BG	LCP	86.21	7.02	22	16	25	Knee stiffness
26	Patient.26	40	M	9573	RTA	Left	Left clavicle fracture	12 days	VI	Bicolumn+BG	LCP	89.99	9.22	28	13	24	
27	Patient.27	45	M	15100	RTA	Right		20 days	V	Bicolumn+BG	Buttress plate	85.51	7.99	23	13	21	Occasional pain
28	Patient.28	34	M	27539	RTA	Right		17 days	VI	Bicolumn	Buttress plate	85.37	7.36	23	14	20	Knee stiffness
29	Patient.29	49	M	56943	RTA	Left		12 days	VI	Bicolumn	Buttress plate	87.64	7.98	25	16	26	Superficial infection
30	Patient.30	60	M	47812	RTA	Right		10 days	V	Bicolumn	Buttress plate	88.31	9.44	27	12	24	
31	Patient.31	57	F	50178	RTA	Left	Left distal radius fracture	16 days	VI	Bicolumn+BG	LCP	86.98	7.22	24	14	23	Occasional pain

## Discussion

High-energy bicondylar tibial plateau fractures (Schatzker types V and VI) represent unstable intra-articular injuries that continue to challenge orthopedic surgeons. The primary goals of operative management are anatomic reduction of fracture fragments, restoration and maintenance of articular congruity, preservation of surrounding soft tissues, and prevention of complications such as infection and malalignment. Several fixation methods have been described in the literature, including external fixation, hybrid external fixation, single lateral locking plate, and dual buttress plate fixation. However, the ideal surgical technique for these complex fractures remains controversial. Single lateral locking plate fixation, often augmented with screws directed medially to hold the medial fragment, offers the advantages of limited soft tissue dissection and percutaneous screw insertion, thereby reducing the risk of wound complications [14]. Nonetheless, this technique has limitations in providing adequate stability in all bicondylar tibial plateau fractures. Failures have been reported particularly in cases involving a medial intra-articular fracture line, small comminuted medial plateau fragments, or coronal fracture components with posteromedial fragments [7].

Studies by Barei et al. and Higgins et al. report the incidence of posteromedial fragments in bicondylar tibial plateau fractures to range between 28.8% and 59% [15,16]. These posteromedial fragments play a crucial role in preventing posterior subluxation of the femoral condyle. A single lateral locking plate may fail to adequately engage the posteromedial fragment, rendering reduction and fixation challenging. Even when lateral plate screws reach the posteromedial fragment, the fixation strength may be insufficient to counteract displacement forces [7,17]. Moreover, due to the fixed-angle design of locking plates, the screws are oriented parallel rather than perpendicular to coronal fracture lines, further compromising fixation in fractures with coronal medial components [7].

Schatzker type V and VI fractures involve both the medial and lateral tibial condyles, necessitating stable reduction and fixation of both columns. The dual plating technique addresses this by stabilizing both medial and lateral columns, thereby restoring mechanical stability with robust fixation [18-23]. Compared to a single lateral locking plate, dual plate fixation demonstrates superior biomechanical strength and a lower rate of subsidence. In a cadaveric biomechanical study, Higgins et al. reported that dual plating resulted in significantly less subsidence than single lateral plating in bicondylar tibial plateau fractures [24]. Additionally, Barei et al. and Yoo et al. have advocated for dual plating, observing favorable functional outcomes in complex tibial plateau fractures [15,25]. However, postoperative malalignment remains a concern. Neogi et al. documented postoperative mal-reduction and malalignment, reporting incidences of 10.9% (three cases) in the single plating group and 6.2% (two cases) in the dual plating group [26]. In our study, postoperative malalignment with delayed loss of reduction occurred in one patient (3.2%), attributed to varus collapse. Despite this, the patient achieved a fair functional outcome at the final follow-up. Mal-reduction on immediate postoperative radiographs was found in four patients (12.9%) in our study, characterized by articular depression with a step-off or gap greater than 2 mm. Of these, three patients had a Modified Rasmussen’s functional score graded as Good, while one patient had a Fair outcome.

One drawback of dual plating fixation is the requirement for extensive soft tissue dissection, which may elevate the risk of wound complications. Various studies have reported the incidence of deep wound infections following dual plate fixation to range from 4.7% to 8.4%. Patil et al. documented a superficial wound infection rate of 2.7% (one patient) in their dual plating cohort [8]. Similarly, Neogi et al. reported a deep infection rate of 3.12%; however, our study did not report any deep infections [26].

Superficial surgical site infections were noted in five patients (16.13%) and were successfully managed with extended antibiotic therapy. Infection remains a common complication following dual plating. Its incidence may be reduced by careful soft tissue handling and delaying surgical intervention for five to six days post-injury to allow resolution of edema and improvement in skin condition. A comparative overview of the present findings with those of previously published studies is provided in Table 3.

**Table 3:** This table summarizes the demographic data, clinical outcomes, complication profiles, and radiological parameters from the present study and compares them with findings from previously published literature Parameters include total number of patients, mean age, follow-up duration, time to weight-bearing, ROM, knee scoring systems (RFS, OKS, and AKSFS), wound complications, malalignment, secondary loss of reduction, fracture healing time, and final angular measurements (MPTA and PPTA). ROM: range of motion; RFS: Rasmussen functional score; OKS: Oxford knee score; AKSFS: American Knee Society Functional Score; MPTA: medial proximal tibial angle; PPTA: posterior proximal tibial angle

	Present study	Cho KY et al. [23]	Prasad et al. [19]	Oh CW et al. [22]	Citak et al. (DP group) [10]
Total number of patients	31	10	40	40	10
Mean age (in years)	49.6	51.6	40	54	51.2
Mean follow-up duration (in weeks)	22.74	33.7	48	25	27.8
Weight-bearing (in weeks)	8	8	12	15	12
ROM	1.5°-130°	0°-122.5°	1.75°-128.5°	0°-123°	0°-119°
Knee score	RFS-25.48	AKSFS-85	OKS: Excellent-30, Good-10	RFS-26	RFS-22.9
Wound complications	5	1	1	2	1
Malalignment	1	2	1	0	1
Secondary loss of reduction	0	1	0	1	0
Healing time (in weeks)	13.74	16	14	19	14
Mean MPTA (at final visit)	87.51°	90.5°	84.0°	Not measured	86.3°
Mean PPTA (at final visit)	8.13°	4.4°	8.25°	Not measured	5.5°
Type of study	Prospective	Retrospective	Retrospective	Prospective	Retrospective

Study limitations

This prospective study involved a limited cohort (n=31) of Schatzker type V and VI bicondylar tibial plateau fractures, with no predefined demographic or fracture pattern criteria. Key limitations include the small sample size, short follow-up duration, and absence of a control group. Further large-scale randomized controlled trials with longer follow-ups are needed to establish standardized treatment guidelines for these complex injuries.

## Conclusions

The surgical management of tibial plateau fractures remains a complex endeavor, with soft tissue evaluation playing a pivotal role in determining the appropriate timing and method of fixation. In this study, dual-column plating through a two-incision approach yielded favorable functional and radiological outcomes, including satisfactory ROM and reliable fracture union. Anatomical restoration of the articular surface was found to be crucial for achieving joint congruity and stability, which are essential for cartilage healing. Our findings support the efficacy of bicolumnar plating in achieving accurate reduction and stable fixation in complex tibial plateau fractures when performed by experienced surgeons. Although early complications such as knee stiffness and superficial infections were observed, these were largely mitigated through the prompt initiation of physiotherapy and meticulous soft tissue handling.
